# Rare Bilateral Variations in the Origin of the Plantaris Muscle: A Case Report and Literature Review

**DOI:** 10.7759/cureus.74668

**Published:** 2024-11-28

**Authors:** Jihad Hawi, Christina El Ahmadieh, Shahd Aboul Hosn, Jad El Masri, Naim Ouaini

**Affiliations:** 1 Anatomy, American University of Beirut, Beirut, LBN; 2 Faculty of Medicine, Lebanese University, Tripoli, LBN; 3 Anatomy, Holy Spirit University of Kaslik, Kaslik, LBN

**Keywords:** anatomical variation, cadaver dissection, case report, plantaris muscle, sural nerve

## Abstract

The plantaris muscle is spindle-shaped in the posterior compartment of the leg. It is distinguished for its small muscle belly and an exceptionally long tendon. It presents with great variability in its origin and insertion when present. This case report presents a novel bilateral variation in the origin of the plantaris muscle in a male cadaver. In addition, it studies the clinical implications behind this novel variation. A 75-year-old male cadaver was dissected following the guidelines of the 16th edition of Grant's Dissector in the Anatomy Lab at the American University of Beirut. This case report showcased that the plantaris muscle in the left lower limb of the male cadaver comprises two separate plantaris muscle bellies that have distinct origins, and their tendons insert distally at the Achilles tendon. While in the right lower limb, two plantaris muscle bellies emerged from the same origin, i.e., the lateral supracondylar line of the femur, and their tendons insert distally at the Achilles tendon. The presence of such an anatomical variation in the plantaris muscle can have significant clinical outcomes that could possibly help physicians with diagnosis and treatment.

## Introduction

The plantaris muscle (PM) is a small muscle of the posterior compartment of the leg that has a short belly and long thin tendon. It normally originates from the lateral supracondylar ridge of the femur and from the oblique popliteal ligament and inserts into the posterior part of the calcaneal tuberosity. Although highly variable in its form and size when present, it can be absent in 5-10% of people. Its significant role in proprioception has been elucidated, especially since it consists of highly dense muscle fibers. When the embryo is 14 mm, muscles of the posterior leg can be separated into two groups: superficial lateral (gastrocnemius, soleus, and PMs) and deep medial (flexor digitorum longus, flexor hallucis longus, popliteus, and tibialis posterior muscles). After two months of embryo development, the two heads of the gastrocnemius muscle appear [1]. The PM would then originate from the lateral head of the gastrocnemius [2]. In mid-term fetuses, the thickness of the PM was found to be similar to that of the lateral head of the gastrocnemius muscle [3]. The insertion of the PM on the plantar aponeurosis plays an important role in the muscle's function to grasp using the foot [4]. The common ancestor of all primates had the insertion of the PM to the plantar aponeurosis. However, with the evolution of humans from quadrupedalism to bipedalism, the PM's function changed. Quadrupedal primates rely on their feet to grasp, but bipedal humans use their feet mainly for posture support. Consequently, the PM was found to be inserted on the calcaneus tuberosity. When the plantar aponeurosis and PM became two separate entities, it permitted the plantar aponeurosis to become stiff, providing support and power during locomotion [5].

Being one of the three muscles forming the superficial posterior compartment of the leg, the PM primarily originates from the lateral supracondylar line of the femur and inserts inferiorly at the posterior surface of the calcaneus bone at the calcaneal tuberosity medial to the calcaneal tendon [6,7]. Meanwhile, the innervation of the PM was discovered to be from the posterior deep crural nerves rather than the superficial nerves suggesting the possibility of the muscle being derived from deeper muscles [8]. In a clinical setting, the PM plays an important role in diagnosing posterior leg pain. It is also involved in differentiating conditions such as PM rupture, non-insertional Achilles tendinopathy, and popliteal artery compression syndrome. The PM tendon has been considered a valuable graft for surgical procedures by several surgical specialties [4]. The morphologically variable PM contributes highly to plantarflexion of the leg and flexion of the knee, making it susceptible to rupture, which is often heard as a pop sound [9]. It is also worth mentioning that variabilities of the plantaris tendon may contribute to the development of Achilles tendinopathy [10].

In this case report, we introduce a new variation of the PM on a male cadaver, which, to our knowledge, has not been reported in the literature. We aim to describe the implications of the variation, highlighting its clinical significance specifically, particularly its diagnostic and surgical aspects.

## Case presentation

**Figure 1:**
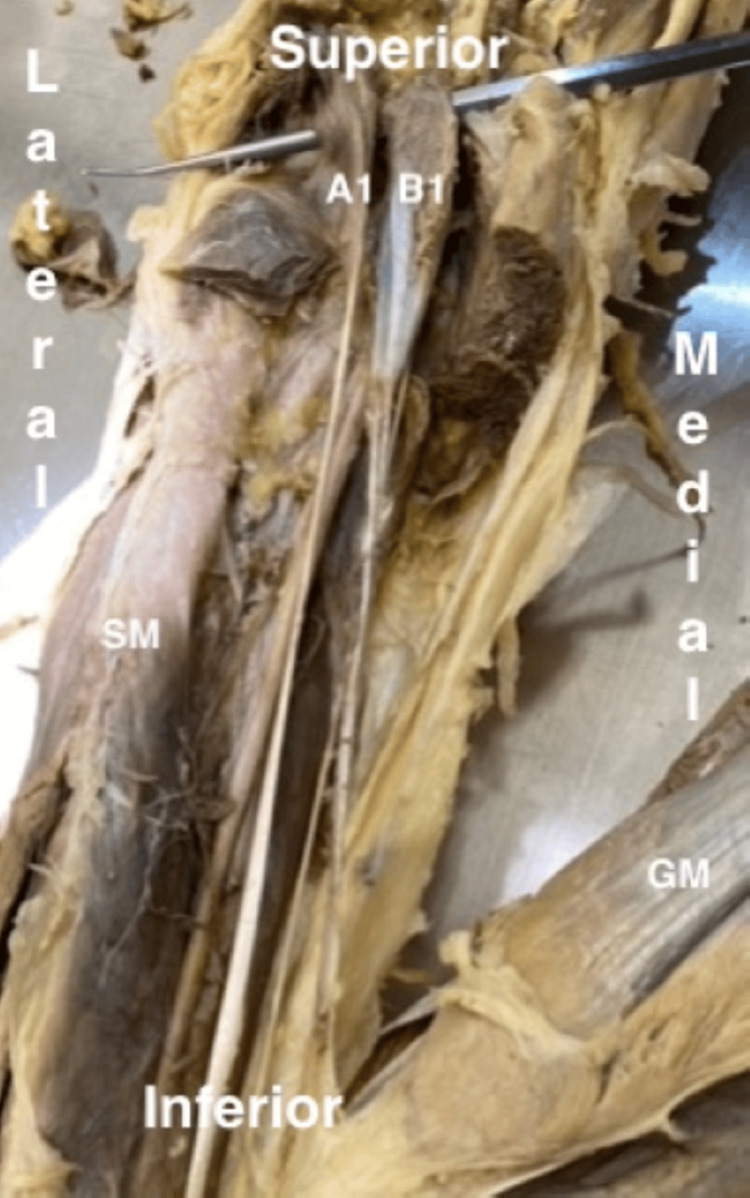
Left leg showing two separate plantaris muscles A1, original plantaris muscle; B1, additional plantaris muscle; GM, gastrocnemius muscle; SM, soleus muscle

**Figure 2:**
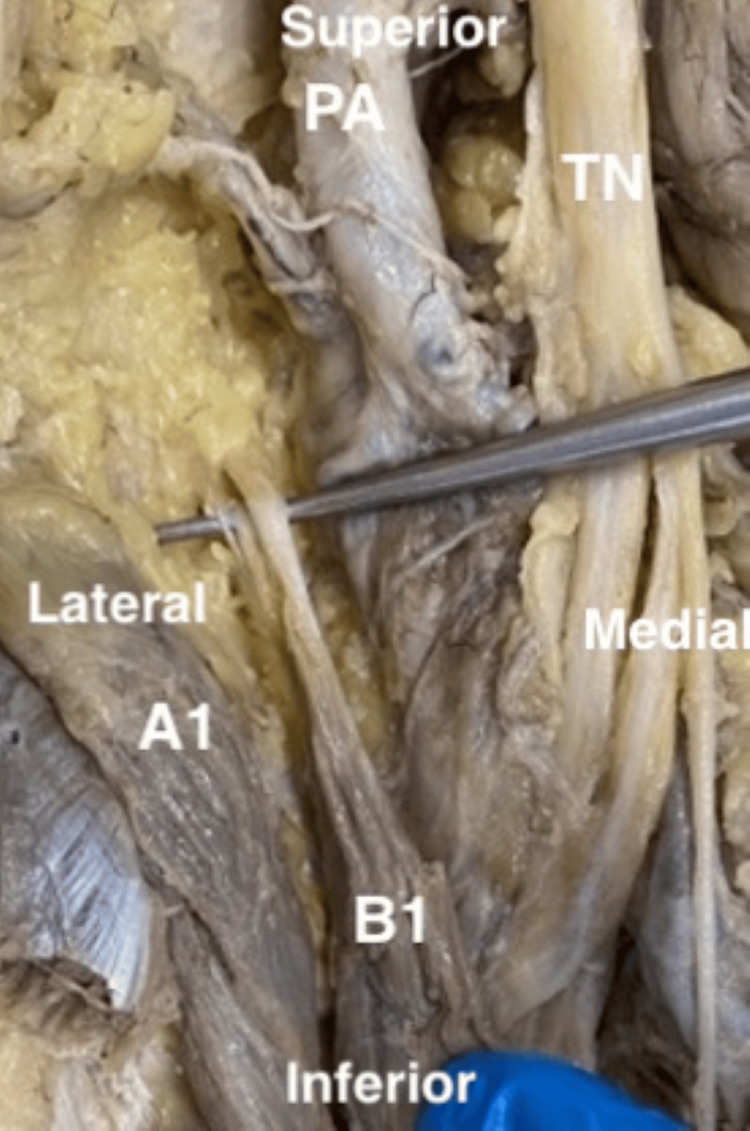
Left leg showing different origins of two plantaris muscles A1, original plantaris muscle; B1, additional plantaris muscle; PA, popliteal artery; TN, tibial nerve

The cadaver of a 75-year-old male was dissected following the 16th edition of Grant’s Dissector by a collaboration of all authors [11]. The dissection was performed in the anatomy lab at the American University of Beirut as part of the education curriculum for first-year medical students. The PM in the left leg appears to have two different bellies with different origins. One of the origins is the lateral supracondylar ridge of the femur bone, denoted as A1 in Figures 1 and 2. This is the most common site of origin for the PM, and the muscle length measures 28 cm. Meanwhile, the second belly, denoted as B1 in Figures 1 and 2, originates distally from the shaft of the femur, measuring 23 cm in length. The two tendons emerge separately following a parallel alignment to reach their common insertion at the calcaneus (Achilles) tendon, which will then insert at the calcaneus bone. Therefore, it is valid to assume that they appear as two distinct muscles, an original PM (A1) and an additional one (B1). 

In the right leg, the PM also originates as two separate muscles, yet in this case, the two muscles have a common origin, which is the lateral supracondylar ridge of the femur. The lengths of the two PMs were similar, i.e., 23 cm. In addition, the two muscles appear to overlap and continue as two independent tendons that insert at the calcaneus tendon distally as shown in Figure 2.

**Figure 3:**
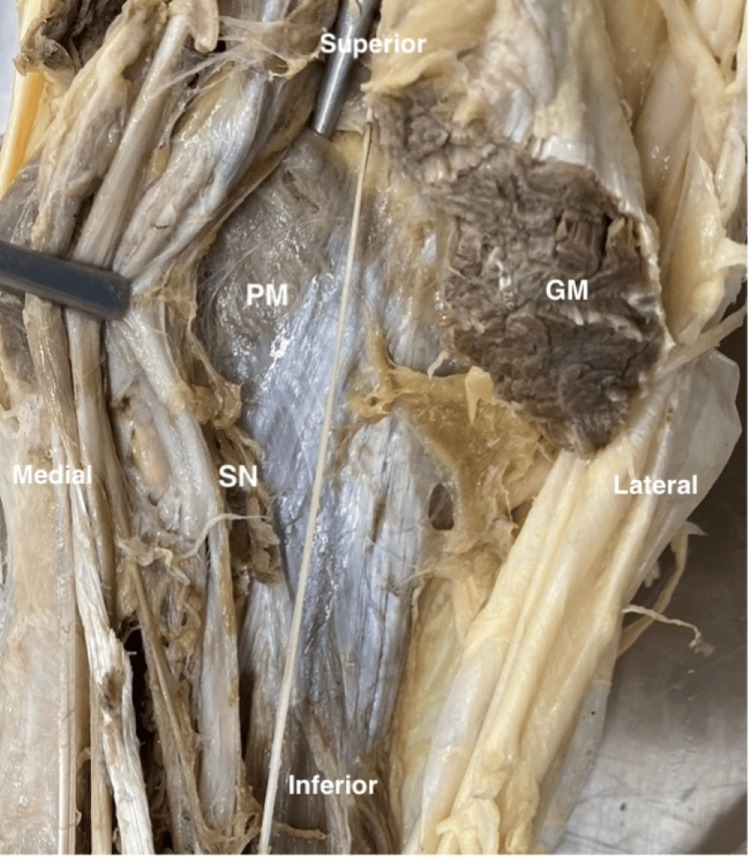
Left leg showing the popliteus muscle The SN is located superficial to the posterior compartment of the leg. GM, gastrocnemius muscle; PM, plantaris muscle; SN, sural nerve

It is important to mention that upon dissection, the popliteus muscle was located deep (anterior) to the PM, and the sural nerve was superficial to the muscle as expected (Figure 3).

## Discussion

In this case study, we present a rare variation of the PM located in the posterior compartment of the lower limb that has not been previously reported. We revealed that the left lower limb comprises two plantaris tendons that emerge from different locations, one from the lateral supracondylar line of the femur and another from the distal posterior shaft of the femur. The PM emerging from the posterior shaft of the femur would be classified as type VI under rare cases [12]. Upon dissection of the right lower limb, we showcased two plantaris tendons similar to the left leg. However, in this case, the muscles originated from the same location, which is the lateral supracondylar line of the femur. It is also worth mentioning that the two separate tendons of the PMs in both legs insert distally at the calcaneal tuberosity. For this reason, we considered the muscles to be two separate entities.

The fusiform normal PM is modified to a biceps muscle by an additional accessory PM belly. The first accessory PM was documented by Hall in 1808 [13]. A study was done on 1,000 MRI exams of the knee to identify the prevalence of accessory PM. This study showed the prevalence of accessory PM to be 6.3%, with an overall prevalence of bilateral PM anatomical variations being around 5% [14]. However, another study conducted on 750 cadavers did not document the presence of any accessory PM [15].

Several variations of the PM have been reported in the literature. One variation revealed a bifurcated PM with two bellies originating separately from the iliotibial tract and lateral supracondylar line of the femur. The superior muscle courses transversely to insert medially at the semimembranosus tendon, while the inferior muscle continues to insert distally at the calcaneal tuberosity [16]. In another study, an interdigitated PM to the lateral head of the gastrocnemius muscle was discovered, and another variant where it forms a sturdy fibrous extension to the patella was analyzed. The latter appears to be involved in the manifestation of the patellofemoral pain syndrome [17]. A case of bicipital origin of the PM was reported, yet the muscle showed a descending path of a single tendon, unlike our case report, which reveals two separate tendons [6]. Similar to this case report, it was reported that two different variant PMs could co-occur in both legs of the individual [18]. Moreover, a study found the plantaris tendon to be completely absent in the right leg. The absence of the PM was discovered to have no functional deficits or implications [19]. Therefore, it can be considered as a vestigial muscle having a more significant sensory role that involves delivering sensory afferent signals to the CNS [20]. Similar to what we presented in this case report, a study displayed an atypical proximal origin of the PM for the first time. In contrast to our study, the two heads of the PMs meet at a more proximal position [21].

The rupture of the PM at its belly or tendon can be classified as a tennis leg, similar to the gastrocnemius muscle rupture. This classification is usually used for gastrocnemius muscle tear in athletes upon simultaneous foot dorsiflexion and knee joint extension. The rupture of PM is accompanied by less pain and a faster recovery when compared to the rupture of gastrocnemius muscle. In addition, the rupture of the medial head of the gastrocnemius muscle is much more common than the PM rupture [22]. In fact, it was reported that an aberrant PM can occlude the popliteal artery resulting in leg pain, especially since a variant plantaris is located more medially [23]. An important clinical role of the PM has been revealed in tendinopathy of the Achilles tendon. The course of the PM tendon and its insertion may contribute to the midportion Achilles tendinopathy, which is mostly found in physically active individuals [24].

Furthermore, the plantaris tendon can be used for grafting and reconstruction in hand surgery when palmaris longus muscle is absent. It is used mainly to stabilize the radioulnar joint. The PM can also be used to reconstruct the Achilles tendon [25]. Similarly, the PM tendon can be a valuable resource as an autograft in concurrent anterior cruciate ligament and anterolateral ligament reconstruction [26]. 

## Conclusions

We presented a case of a 75-year-old male cadaver of a new variation of the PM that appeared to emerge as two separate muscle tendons in both legs, inserting both at the calcaneal tuberosity. To our knowledge, such a variation has not been reported before. Clinicians must be aware of such anatomical variations in the PM, as it can have several clinical complications. Further anatomical studies and imaging modalities must be employed to reveal the prevalence, morphology, and clinical relevance of this rare variant of PM.
